# New Isolated Shrimp (*Litopenaeus setiferus*) Chitosan-Based Films Loaded with Fly Ash for Antibacterial Evaluation

**DOI:** 10.3390/polym14102099

**Published:** 2022-05-21

**Authors:** Seham S. Alterary, Narguess H. Marei

**Affiliations:** Department of Organic Chemistry, College of Science, King Saud University, P.O. Box 50013, Riyadh 11523, Saudi Arabia; narguess.hm@gmail.com

**Keywords:** chitin isolation, chitosan, fly ash, deacetylation, antibacterial activity

## Abstract

New three fabricated chitosan (CS) loaded with fly ash (FA) films were developed in this study. The shell waste of white shrimp was used as a precursor for the isolation of chitin and converted into chitosan by carrying out a deacetylation process. The formation of chitosan was conducted by various preparation steps deproteinization, demineralization, and deacetylation. The degree of deacetylation was found to be 95.2%. The obtained chitosan was used to prepare three different chitosan loaded-fly ash films. The prepared films contained various fly ash: chitosan ratios (2:1, FA-CSF1), (1:1, FA-CSF2), and (1:2, FA-CSF3). The obtained films were characterized using FTIR, XRD, and SEM. The micrograph images of the formed films showed spherical particles with an average size of 10 µm. The surface area, adsorption-desorption properties, thermal stability, and water/fat binding features of the fabricated chitosan films were studied. The results revealed that the prepared films displayed typical BET graphs with surface areas ranging from 2.436 m^2^ g^−1^ to 8.490 m^2^ g^−1^. The fabricated FA-CSF films also showed high thermal stability at temperatures up to 284.9 °C and excellent water/fat binding capacities. The antibacterial potential of the designed films was screened against *E. coli* (Gram-negative) and *B. cereus* (Gram-positive) bacterial strains. The tested solution of CS (1%) exhibited inhibition zones for *E. coli* and *B. cereus* as 18.51 mm and 14.81 mm, respectively, while in FA solution (1%), the inhibition zones were found to be 10.16 mm, and 13.57 mm, respectively. The results encourage and open up the new and promising areas of research for applying chitosan extracted from waste materials in biological applications.

## 1. Introduction

*Litopenaeus setiferus* (White shrimp) has become one of the most significant aquaculture shrimps in the world. It has a wide range of salt tolerance, quick growth, and other features appropriate for intensive aquaculture [1]. Shrimps are small marine crustaceans that are widely consumed as shellfish. Because of their high vitamin and mineral content, such as copper, calcium, zinc, iron, and phosphorus, as well as their high amino acid and protein content, they constitute a typically nutritious meal [2]. The biochemical composition of shrimps is highly influenced by various factors including, species, size, age, natural diet, feed composition, and environmental factors [3]. Shrimps have a seasonal shift in their fatty acid profile, cholesterol, and total carotenoid concentration [4,5]. Their physicochemical, proximate composition, microbiological, and sensory qualities, on the other hand, have received little attention in relation to their origin and treatment [6]. 

Chitin is the world’s second most prevalent polysaccharide after cellulose and represents the main component in the exoskeleton of arthropods such as insects, and crustaceans. It is present in green algae, fungi’s cell walls, insect and spider cuticles, and in the crustacean’s exoskeletons [7]. The leftover residues from crustacean (shrimp, crab, lobster, and prawn) processing factories are the principal source of chitin on an industrial scale. The majority of crustacean shells are composed of chitin (15–40%), protein (20–40%), calcium and magnesium carbonate (20–50%), as well as other minor elements such astaxanthin, lipids, and other minerals [8]. The isolation strategies documented are varied, as they are mostly dependent on the composition of the source, which varies greatly from one species to the next [9]. The majority of these methods use chemical processes to extract the protein and remove the inorganic debris. Some of them include a solvent extraction or oxidation stage to bleach the residual pigments [10]. Because of increased intramolecular and intermolecular hydrogen bonding, chitin is insoluble [11]. The soluble form of chitin is chitosan which is obtained by deacetylation of chitin. Chitosan is an abundant naturally occurring polymeric polysaccharide comprised of poly [-(1,4)-2-amino-2-deoxy-D-glucopyranose]. The percentage of D-glucosamine units compared to the sum of acetylated and deacetylated units in the molecule is represented by the degree of deacetylation (DD) of chitosan. Because of its versatility and desired qualities, such as biodegradability, biocompatibility, low toxicity, great availability, and low cost, it has gained a lot of attention [12]. 

Chitin is also the most second prevalent biopolymer after cellulose, and it is recognized for its efficient biodegradability, biocompatibility [13], non-immunogenicity [14], and muco-adhesiveness [15]. It is generally recognized as the “safest polymer” by Food and Drug Administration United States [16]. 

Chitosan has been reported to possess various biological properties including, antimicrobial, anticancer, anti-inflammatory, hemostasis, and wound healing [17,18,19,20,21]. It has also been widely utilized for food packaging [22], drug delivery [23], and wound dressing [24] applications. Chitosan has displayed remarkable film coating abilities with an excellent gas barrier, improved permeability of water vapor, and protection from UV light [25]. It has a promising ability to encapsulate therapeutic drugs and release them over time in a regulated manner [26]. In addition, its cationic feature facilitates effective polyelectrolyte interactions and ionic linkages with multivalent anions, which makes it easy to derivatize or functionalize [27]. Different approaches have been applied to prepare chitosan-based nanostructures, including nanoprecipitation, ionic gelation, reverse micellization, covalent cross-linking, and emulsion solvent evaporation [28,29,30,31,32]. Recently, diverse natural products (plants, sponges, cyanobacteria, plant wastes, bacteria, and crustaceans) have been encapsulated with chitosan to boost their efficiency and therapeutic properties [33,34,35]. 

Fly ash (FA) is the finest coal combustion product produced after pulverized coal is burned to create electricity. It is made up of small inorganic mineral particles with a minor amount of carbon. Because of the high silica content, it usually has a crystalline appearance. Therefore, it is a cost-effective cement addition that can also be used to stabilize geotechnical engineering that requires loose soils. The most prevalent type of fly ash is made up of crystalline materials such as quartz, glassy sillimanite (Al_2_SiO_5_), and mullite (Al_6_Si_2_O_13_) [36]. Fly ash has been reported for various applications such as agriculture and environmental protection [37], remediation of water contaminations [38], heat transfer applications [39], dye removal [40], and pathogen inactivation [41]. 

The objective of the present study is the isolation of chitosan from the white shrimp shells by deacetylation of chitin. The isolated chitosan was employed to fabricate chitosan-based films loaded with fly ash. The fabricated films were characterized using FT-IR, XRD, and SEM analyses. The increase in the concentration of chitosan in the fabricated films was tested against two bacterial strains *Bacillus cereus* (Gram-positive) and *Escherichia coli* (Gram-negative) to investigate the inhibition potential in their growth.

## 2. Materials and Methods

### 2.1. Chemicals and Reagents 

Sodium hydroxide of purity (99.9%), hydrochloric acid (36.5%), ethanol (95%), acetone (≥99.5%), and glacial acetic acid (≥99.7%), were purchased from Sigma-Aldrich (Hamburg, Germany). Fly ash consists of oxides of aluminum, silicon, iron and calcium, magnesium, potassium, sodium, titanium, and sulfur, and was obtained from power plants, College of Science, King Saud University, Riyadh, Saudi Arabia. White shrimp (*Litopenaeus setiferus)* shells, sunflower oil, and olive oil was purchased from the local markets in Riyadh, Saudi Arabia.

### 2.2. Bacterial Strains and Maintaining Culture

Two bacterial strains, *Bacillus cereus* (*B. cereus*, Gram-positive, ARCC14579) and *Escherichia coli* (*E. coli*, Gram-negative, ATCC25966) were obtained from the Microbiology Department, College of Science, King Saud University, Saudi Arabia. The test strains were cultured in Mueller–Hinton broth (Sigma-Aldrich, Hamburg, Germany) and incubated at 37 °C overnight in a Thermo Scientific^TM^ MaxQ^TM^ 4000 Benchtop Orbital Shakers. Using a UV spectrophotometer Thermo Scientific^TM^ Orion AquaMate 8000, the optical density (OD) was optimized to 0.1 at λ = 625 nm. 

### 2.3. Isolation of Chitin from White Shrimp Shells 

**Scheme 1:**
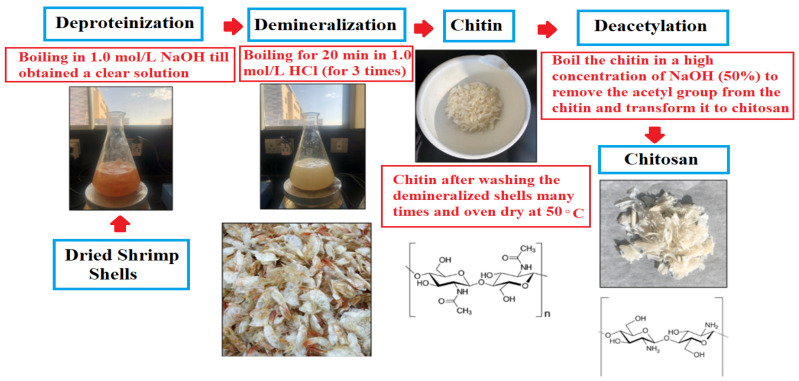
Schematic illustration described the isolation steps of chitin from white shrimp shells and the production of chitosan by deacetylation of the isolated chitin.

White shrimp shells were used to isolate chitin. The separation process was conducted in four conventional steps including demineralization, deproteinization, decolorization, and deacetylation using the previously standard reported method [42]. Briefly, the shells of shrimps were scraped from the loose tissues, collected, and washed thoroughly with tap water to eliminate any contaminants. The cleaned shells were air-dried and blended into small pieces of less than 20 mesh. The deproteinization step was carried out using 100 g of the homogenized shells with 1.0 mol L^−1^ sodium hydroxide at 100 °C for 8 h. The demineralization step was performed by treating the same quantity of homogenized shells with 1.0 mol L^−1^ of hydrochloric acid (1500 mL). The pH of the resulting solid material was neutralized using distilled water. The isolated chitin was neutralized using distilled water. The isolation process was performed in triplicates. The isolated chitin was then rinsed using ethanol followed by acetone to eliminate any remaining contaminants. The purified chitin was dried to a consistent weight in a vacuum oven for 2 h at 50 °C. The deacetylation step to convert chitin to chitosan was generally conducted by treating the chitin sample with concentrated (40–50%, w/v) sodium hydroxide at 100 °C for 30 min. The resulting chitosan was filtered, oven-dried, and stored in a dissector for further experiments (Scheme 1).

### 2.4. Fabrication of Chitosan-Based Films Loaded with Fly Ash

Chitosan-based films loaded with fly ash were prepared with different ratios of FA and CS. Three different chitosan-loaded fly ash (FA-CS) films were fabricated using approximately 0.5 g of chitosan dissolved in 1% (*v*/*v*) acetic acid under agitation for 5 min, then 1.0 g of fly ash was added to prepare FA: CS concentration ratio of 2:1 (FA-CSF1), 0.5 g of fly ash was added to 0.5 g CS to prepare 1:1 (FA-CSF2), and 0.5 FA: 1.0 g CS was used to prepare 1:2 (FA-CSF3) films, respectively. The suspended mixtures were poured into a rounded flat container and incubated overnight at 50 °C [43].

### 2.5. Characterization of the Fabricated FA-CS Films

The fabricated chitosan-loaded fly ash films were characterized using various spectroscopic and microscopic techniques. A Thermo Nicolet Avatar-360 ESP FTIR spectrophotometer (Thermo Scientific™, Waltham, MA, USA) was used in a wavenumber range of 4000–400 cm^−1^ to scan and determine the functional groups present in the as-prepared films. X-ray diffraction (XRD) Bruker D5005 diffractometer (Bruker™, Billerica, MA, USA) was applied using Cu Kα radiation (45 kV, 30 mA), 2θ = 5–65° at a scan rate of 4 min^−1^, and at temperature of 25 °C to detect the crystallinity of the films [44]. A scanning electron microscope (SEM JEOL JSM-6300, Billerica, MA, USA) was used to determine the morphology differences between the fabricated films. The samples were gold-sputtered prior to examination. The electron beam was with 10 kV energy and magnifications of ×2000 [45].

### 2.6. Thermogravimetric Analysis 

The thermal properties of the fabricated chitosan films were studied. Thermogravimetric analysis (TGA) was utilized to measure the thermal stability and to determine the dedradation temperature of dried film powder, using a VersaTherm© Thermogravimetric Analyzer (Cahn Scientific, Irvine, CA, USA). The mass of each sample was ranged from 5 to 6 mg. The temperature was optimized from 25 to 800 °C with a heating rate (10 °C/min) and maintained in isotherm for 5 min under a nitrogen gas condition with a flow rate of 50 mL min^−1^. The mass of the sample was continuously recorded as a function of temperature. The degradation percentage and temperatures were estimated in triplicates from the percentage weight loss first derivative graph (DTGA) against temperature [46].

### 2.7. Brunauer–Emmett–Teller Surface-Area Analysis

The surface area and porosity of the fabricated chitosan (CSF), as well as chitosan loaded with fly-ash (FA-CSF) films, were evaluated using the Brunauer–Emmett–Teller (BET) method. A Micromeritics Gemini 2360 surface area analyzer (Norcross, GA, USA) was used to carry out the measurements. The chitosan films were degassed at 25 °C before testing. The adsorption-desorption of nitrogen gas molecules were carried out based on the standard pressure range (0.05 ˂ P/Po ˂ 0.3) and the occupied area by nitrogen molecule 16.2 Å^2^ at 77.35 K.

### 2.8. Water and Oil Binding Capacity

The water and fat binding capacity of the fabricated chitosan films were studied by weighing a centrifuge tube of 15 mL containing 50 mg of each sample. The samples were dispersed by adding 4.0 mL of distilled water and vortexing for 1 min at 3400 rpm. The last step was repeated by adding approximately 4.0 mL of olive oil and sunflower oil. The ingredients were left at room temperature for 1 h. After that, the samples were centrifuged for 30 min at 6000 rpm. The tubes were weighed after the supernatant was discarded. Triplicates of each sample were examined [47].

The water-binding capacity (WBC) was estimated as follows:

WBC% = (Water bound (g)/Sample Weight (g)) × 100(1)

The oil binding capacity (OBC) was estimated as follows:

OBC% = (oil bound (g)/Sample Weight (g)) × 100(2)

### 2.9. Degree of Deacetylation Determination

The degree of deacetylation was measured by dissolving 0.5 g dried CS in 20 mL of 0.1 mol L^−1^ hydrochloric acid and 25 mL deionized water under continual stirring for 30 min. Stirring was continued for another 30 min after adding another 25 mL deionized water. The solution was titrated with a 0.1 mol L^−1^ sodium hydroxide solution using an automated burette once the chitosan was completely dissolved (0.01 mL accuracy). The degree of chitosan deacetylation (DD) was estimated using the formula:

DD% = 2.03 × (V2 − V1)/(m + 0.0042 (V2 − V1))(3)

where m expressed the weight of the sample (0.5 g CS), V1 and V2 represent volumes of NaOH (0.1 mol L^−1^) corresponding to the deflection points. The values 2.03 and 0.0042 expressed the coefficient resulting from the molecular weight of chitin monomer unit and that resulting from Chitin-chitosan molecular weight difference. 

### 2.10. Antibacterial Activity 

The antibacterial potential of the FA, CS, and the fabricated (FA-CSF) films was studied against two bacterial strains *B. cereus* and *E. coli* using the agar diffusion method. The Mueller–Hinton agar plates were inoculated with the two bacterial strains (100 µL of density 1 × 10^6^ CFU/mL) and incubated for 24 h at 37 °C. Prior to the inclusion of bacteria, the test samples were sterilized in a biosafety cabinet that was dosed with UV radiation for 1 h. In the shaking incubator, sterilized samples were cultured with bacterial strains overnight at 37 °C. The experiment was done in triplicates, and the inhibition zones were measured in millimeters (mm) by measuring the diameter of the zone with no bacterial growth [48].

### 2.11. Statistical Analysis

The data were presented in the form of means and standard deviations (SD). To determine statistical significance, ANOVA test using Excel of Microsoft Office V 10.0.0 was used to estimate the variance, followed by the student’s *t*-test. The significance level was set at a *p*-value of ˂ 0.05.

## 3. Results and Discussion

The isolation process of chitin from shells of white shrimp waste can be performed in many stages. The two critical steps were the deproteinization and demineralization. In this study, the removal of protein was carried out using sodium hydroxide prior to the demineralization to control the formation of foam during the treatment process and hence, could optimize the yield of chitin [49]. The demineralization step was successfully accomplished by treating the shrimp waste with 1.0 mol L^−^^1^ hydrochloric acid solution to dissolve calcium carbonate and remove other organic matters. The obtained residue was chitin. Meanwhile, chitosan is the deacetylated product of chitin. Therefore, the deacetylation degree of the isolated chitin to obtain chitosan was studied and the results revealed a high deacetylation degree with a value 95.2%.

### 3.1. Determination of Degree of Deacetylation 

A simple titration method was used to determine the degree of deacetylation of the isolated chitosan. The titration analysis was based on the titration of chitosan solution dissolved in 0.1 mol L^−1^ hydrochloric acid and titrated with 0.1 mol L^−1^ of sodium hydroxide and from the two inflection points (V1 and V2) corresponding to the acid consumed by the amine groups. The calculated deacetylation value was found to be 95.2%, which is consistent with the results reported in previous studies [50].

### 3.2. Characterization of the Fabricated FA-CS Films

**Figure 1:**
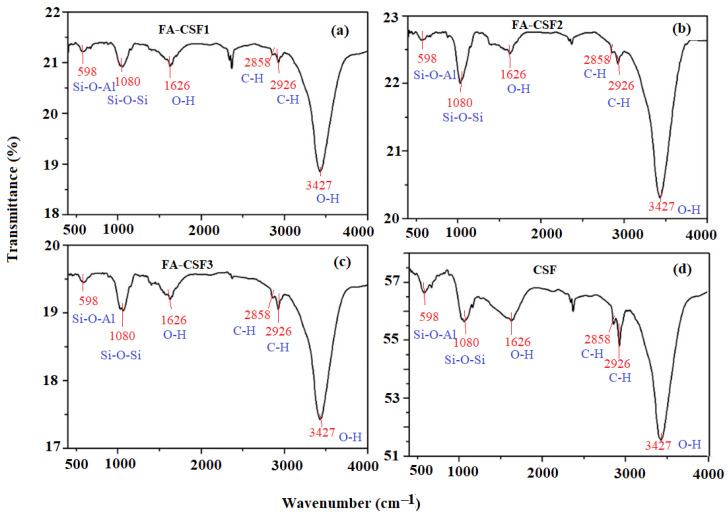
Fourier transform infrared spectra for chitosan-based film (CSF) and fly ash loaded chitosan-based film (FA-CSF), where (**a**) FA-CSF1, (**b**) FA-CSF2, (**c**) FA-CSF3, and (**d**) CSF.

FTIR analysis was used to characterize the functional groups of the prepared chitosan-based films loaded with fly ash (Figure 1). It was observed that all samples showed a strong band at about 3427 cm^−1^ corresponding to OH stretching vibration and asymmetric stretching vibration of primary amine (–NH_2_). The symmetric and asymmetric C-H stretching vibration peaks of CSF were recorded at 2926 and 2858 cm^−1^, respectively. In all samples, a significant band at 1626 cm^−1^ was observed corresponding to residual N-H bending vibration group obtained from primary amine [51], and bending O-H vibration group suggesting the presence of molecular water [36]. These bands are typical of polysaccharides, which are found not only in CS but also in Xylan, carrageenan, and glucan. A band at about 1080 cm^−1^ was found in the Chitosan-loaded fly ash samples (FA-CSF1, FA-CSF2, and FA-CSF3) due to Si-O-Si or Si-O-Al asymmetric stretching vibration. In addition to this, at approximately 598 cm^−1^, a band appears in FA-CSF1, FA-CSF2, and FA-CSF3 suggesting Si-O-Al vibration [52,53,54,55]. 

**Figure 2:**
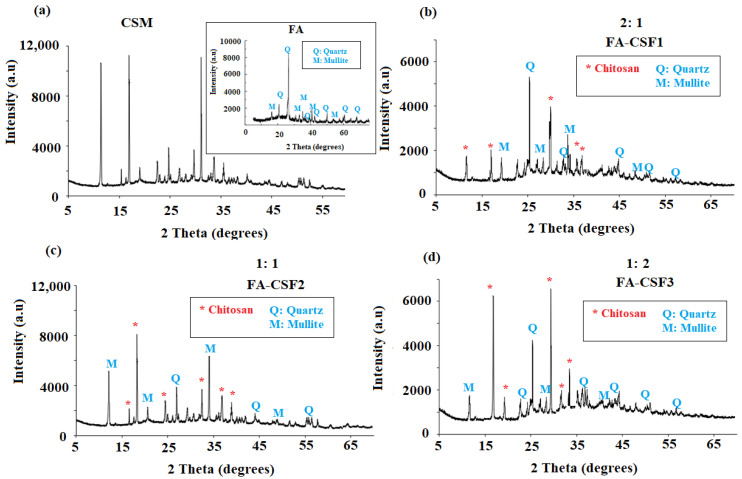
X-ray diffraction for (**a**) chitosan-based film (CSF) and (**b**) fly ash loaded chitosan-based CSF-FA1, (**c**) FA-CSF2, and (**d**) FA-CSF3 films.

The XRD analysis was used to study the crystalline phases of FA, CSM, FA-CSF1, FA-CSF2, and FA-CSF3. The XRD pattern of the FA showed two main crystalline phases Quartz and Mullite. The large hump at 2θ at 25–30° indicated the amorphous phases of the fly ash [56]. The early studies revealed that the Al_2_O_3_, SiO_2_, and Fe_2_O_3_ are the main oxides present in the FA and represent approximately 70% of the total mass (Figure 2a). The XRD pattern of CS-based film displayed three main 2θ position peaks at 10°, 20° and 31° indicating the presence of the crystalline phase of the chitosan [57]. The XRD patterns of the FA-CSF1, FA-CSF2, and FA-CSF3 films showed the presence of the crystalline phases of chitosan and FA indicating well loading of FA on the chitosan surface (Figure 2b–d).

**Figure 3:**
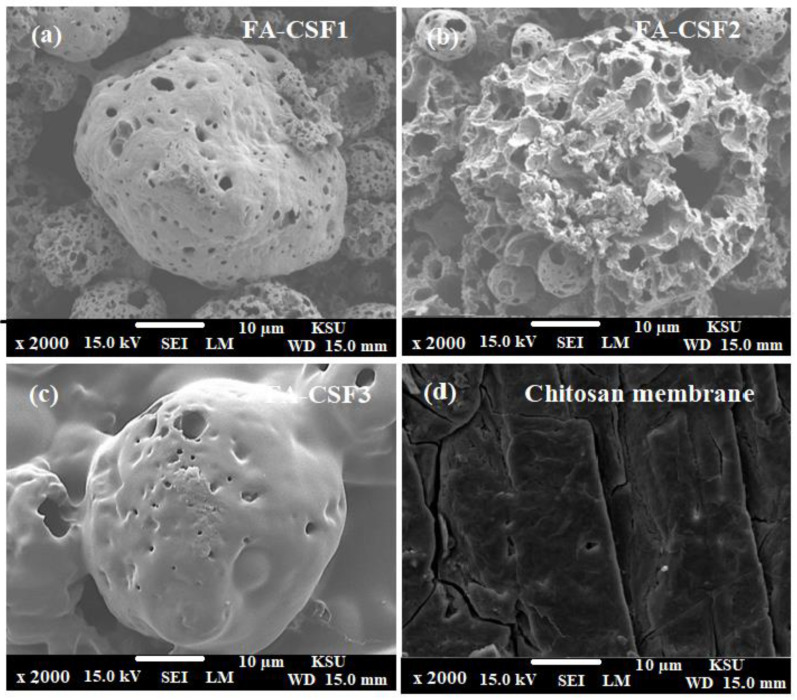
Scanning electron micrographs (SEM) for (**a**) FA-CSF1, (**b**) FA-CSF2, (**c**) FA-CSF3, and (**d**) Chitosan-based membrane (CSF) with a magnification ×2000.

The surface morphology and effect of fly ash loading on the chitosan-based films CSF, FA-CSF1, FA-CSF2, and FA-CSF3 were studied by SEM. Microspheres comprise the majority of FA particles. FA particles were found in two different spherical forms (Figure 3). It is possible to detect hollow spheres (cenospheres) and thin-walled hollow microspheres with smaller ones enveloped or minerals within these spheres called plerospheres [58]. Furthermore, the smoothness of the microsphere and the clearer surfaces were evident after combining chitosan with fly ash, suggesting the incorporation of the CS with FA (Figure 3a–c).

### 3.3. Thermogravimetric Analysis 

**Figure 4:**
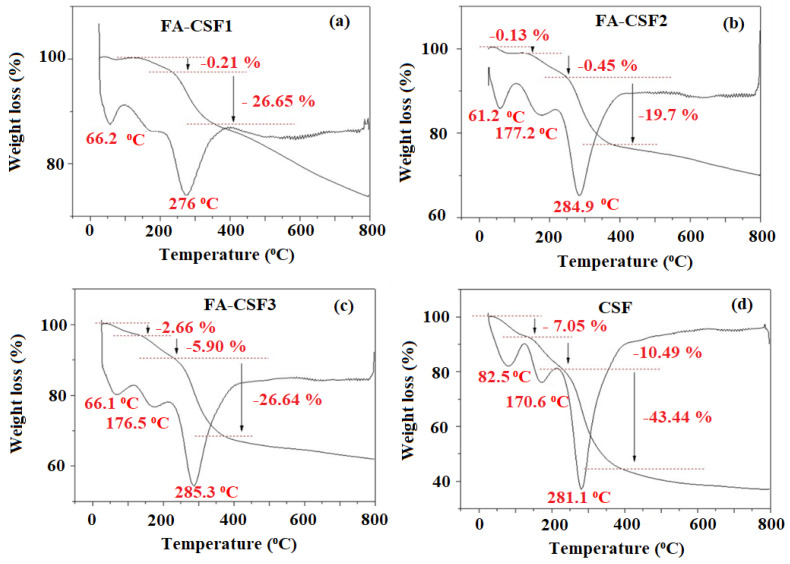
Thermogravimetric analysis showing the effect of fly ash concentration on the thermal stability of chitosan membrane, where (**a**) FA-CSF1, (**b**) FA-CSF2, (**c**) FA-CSF3, and (**d**) CSF.

TGA analysis allows continuous monitoring of the mass of the samples as a function of temperature and time, while an inert gas atmosphere was passed over the samples. A dynamic TGA was performed for CSF, FA-CSF1, FA-CSF2, and FA-CSF3. The results showed that in the FA-CSF1, fly ash loaded chitosan-based films with a ratio of 2:1, an initial weight loss of 0.21%, followed by 26.65% with a decomposition temperature of 66.2 °C, and 276 °C (Figure 4a). Moreover, in the FA-CSF2, fly ash loaded chitosan-based films with a ratio of 1:1, an initial weight loss of 0.13%, followed by 0.45%, and 19.7% with a decomposition temperature of 61.2 °C, 177.2 °C, and 284.9 °C (Figure 4b). Additionally, FA-CSF3, fly ash loaded chitosan-based films with a ratio of 1:2, an initial weight loss of 2.66%, followed by 5.90%, and 26.64% with a decomposition temperature of 61.2 °C, 177.2 °C, and 284.9 °C (Figure 4c). Finally, the CSF, chitosan-based films, an initial weight loss of 7.05%, followed by 10.49%, and 43.44% with a decomposition temperature of 82.5 °C, 170.6 °C, and 281.1 °C (Figure 4d). The outcomes revealed that all samples showed decomposition temperatures that have matched those obtained by chitosan-based films [59,60].

### 3.4. Brunauer–Emmett–Teller (BET) Surface-Area Analysis

**Figure 5:**
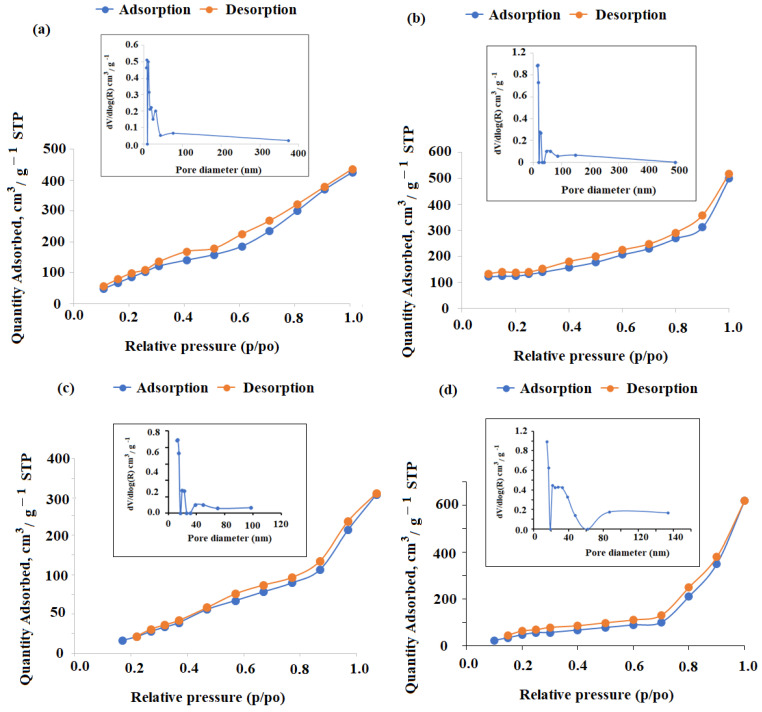
BET graphs of adsorption-desorption of N_2_ gas on the surface of fly ash loaded chitosan (**a**) CSF, (**b**) FA-CSF1, (**c**) FA-CSF2, and (**d**) FA-CSF3 films and their pores diameter.

The surface area and adsorption-desorption properties of the fabricated CSF, FA-CSF1, FA-CSF2, and FA-CSF3 films were investigated using BET analysis. The results showed that typical-BET isotherm graphs were obtained. At low pressure (P/Po ˂ 0.8) relatively flat adsorption isotherms were recorded, while at high pressure (P/Po ˃ 0.8) the adsorption isotherms increased rapidly (Figure 5a–d). Moreover, the largest surface area was achieved when the CS and FA concentrations were equal in the FA-CSF2 with 8.490 m^2^ g^−1^ followed by the CSF with 8.058 m^2^ g^−1^. By increasing FA concentration, the surface area decreases to 7.396, and 2.436 m^2^ g^−1^, in the FA-CSF1, and FA-CSF3, respectively.

The Barrett–Joyner–Halenda (BJH) pore size distribution graphs showed that the obtained pore volume values for adsorption-desorption were CSF (0.034, 0.033 cc/g), FA-CSF1(0.014, 0.017 cc/g), (0.033, 0.035 cc/g), and (0.011, 0.012 cc/g), and average pore radius (15.283, 17.065 Å), (24.452, 24.395 Å), (15.295, 15.265), and (17.067, 17.065 Å) for the above-mentioned chitosan films, respectively. The decrease in the pore volume of the FA-CSF3 film could be attributed to the block effect of a high FA loading concentration in the film.

### 3.5. Water and Oil Binding Capacity

Water and fat binding capacities using sunflower oil and olive oil for the CSF, FA-CSF1, FA-CSF2, and FA-CSF3 were evaluated, and the results were summarized in Table 1. The results showed that the loading of the chitosan-based films with fly ash increased the WBC ranging from 305.24% to 1725.24%. These could be attributed to the dissimilarity in the chitosan crystallinity, number of salt-forming functional groups, and the residual protein content after isolation. Meanwhile, OBC is one of the most crucial properties of chitosan that estimates its usefulness for various applications. Previous studies [61] addressed that the FBC is directly influenced by the deproteinization and demineralization steps. The FBC was performed for all CS films using sunflower oil and olive oil. The results showed that increasing the FA concentration in relation to the decrease in CS and the OBC of CSF in olive oil was decreased from 753.52% to 584.99%, whereas increasing the FA concentration increased the OBC in sunflower oil. The WBC and OBC of the chitosan-based film were consistent with the previously reported results [62]. 

### 3.6. Antibacterial Activity 

Chitosan was known for its wide range of antibacterial activity against a broad range of microorganisms. Many mechanisms have been proposed for the action of chitosan on the bacterial cell, but the most meaningful mechanism is the polymer backbone charged groups and their ionic interactions with the constituents of the bacterial cell wall leading to the hydrolysis of the peptidoglycans in the bacterial cell wall, allowing the leakage of intracellular [63]. 

**Figure 6:**
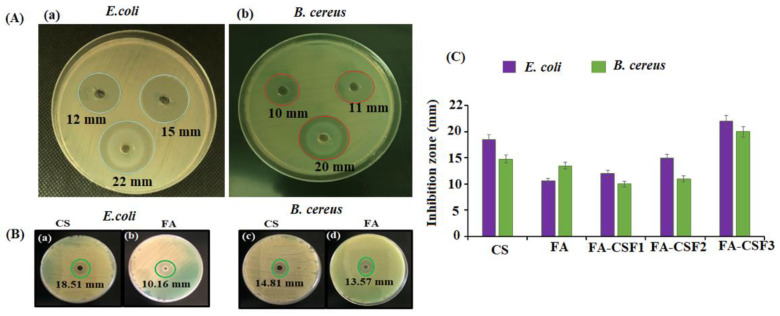
(**A**(**a**,**b**)) the inhibition zones displayed by FA-CSF1, FA-CSF2, and FA-CSF3 films on *E. coli* and *B. cereus*, respectively. (**B**(**a**,**b**)) Inhibition zone diameter of 1% CS and 1% FA solutions against *E. coli* and (**B**(**c**,**d**)) against *B. cereus*, respectively. (**C**) Zone of inhibition diameter/mm, Data were presented in average mean ± SD (*n* ≥ 3).

The antibacterial activity of CS (1%), FA (1%) solutions, FA-CSF1, FA-CSF2, and FACSF3 films displayed varying degrees of sensitivity against *E. coli* and *B. cereus*. Collectively, both bacterial microorganisms were sensitive to the tested samples. The results showed that the fabricated films exhibited potent antibacterial activities against *E. coli* and *B. cereus*, followed by CS and FA. The strongest antibacterial effect of FA-CSF films was towards *E. coli*. The results showed inhibition zones (12, 15 and 22 mm), (10, 11 and 20 mm) for FA-CSF1, FA-CSF2, and FACSF3 against *E. coli* and *B. cereus*, respectively (Figure 6A). Meanwhile, the CS and FA displayed antibacterial activity against both types of bacterial cells [36]. The CS (1%) solution showed a bacterial inhibition zone diameter larger than FA on both *E. coli* and *B. cereus* (Figure 6B). The CS inhibition zones on *E. coli* and *B. cereus* were 18.51 mm and 14.81 mm, respectively, while in FA the inhibition zones were 10.16 mm, and 13.57 mm, respectively [64]. 

**Figure 7:**
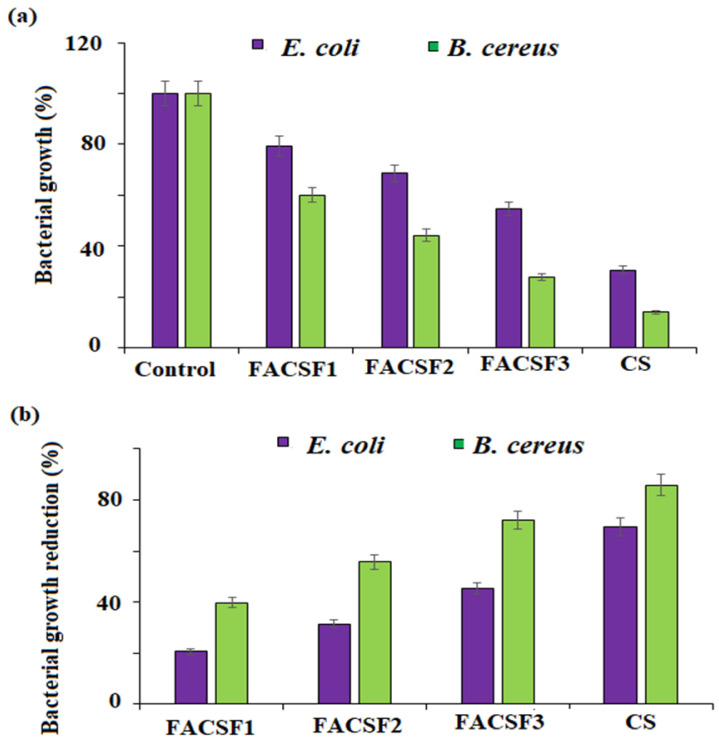
(**a**) Bacterial growth %, and (**b**) bacterial growth reduction % against *E. coli* (Gram-negative) and *B. cereus* (Gram-positive) microorganisms. Data were presented in average mean ± SD (*n* ≥ 3).

Furthermore, the bacterial growth % and bacterial growth reduction % were determined. The bacterial growth % was compared with the bacterial cell growth without the addition of any sample (control) where the growth % was considered 100%. The results revealed that the growth % was the lowest with the CSF. The % growth rate was 30.56%, 54.62%, 68.52%, and 79.28% with the *E. coli*, and 14.04%, 27.77%, 44.29%, and 60.13% with the *B. cereus*, using CSF, FA-CSF3, FA-CSF2, and FA-CSF1, respectively (Figure 7a). The results showed that by decreasing the fly ash-loaded concentration with CS, bacterial growth was inhibited and decreased. This was also visualized in Figure 7b by representing the samples by subtracting the bacterial growth % from 100. It was reported that purified FA reduced the bacterial growth of *E. coli* by 59.89%. However, the FA reduced the growth of the tested bacteria by only 4.95%.

## 4. Conclusions

The present study describes the isolation process of chitin from the shells of white shrimp (*Litopenaeus setiferus*) waste. The isolation procedure was conducted using different steps including, deproteinization, demineralization, and deacetylation to obtain chitosan. The deacetylation degree of the isolated chitin to obtain chitosan was studied and the results revealed a high deacetylation degree with a value 95.2%. The isolated chitosan was loaded with different percentages of fly ash and used to fabricate three FA-CSF films. The physical and chemical properties of the fabricated chitosan films were studied using various spectroscopic and microscopic techniques such as FTIR, XRD, and SEM. Moreover, the thermal stability, the adsorption-desorption, surface area, and the water/oil binding features of the fabricated films were also studied. The results revealed high stability of the fabricated films with excellent water/oil binding capacities. The antibacterial potential of 1% CS and FA solutions and the fabricated FA-CSF films were screened against two bacterial strains *E. coli* and *B. cereus*. The fabricated FA-CSF3 showed the largest zone of inhibition against *E. coli* stains compared to *B. cereus* and others. The outcomes of the current study open up a promising view in the future to exploit the isolated chitosan from shrimp waste to be applied in various biomedical applications.

## Figures and Tables

**Table 1:** The water binding capacity (WBC)%, and oil binding capacity (OBC)% of chitosan-based films loaded with fly ash.

Sample	Water Binding Capacity (WBC)%	Fat Binding Capacity (OBC)%	
		Olive Oil	Sunflower Oil
FA-CSF1	1725 ± 0.04	1090 ± 0.04	709.7 ± 0.01
FA-CSF2	718.5 ± 0.01	611.3 ± 0.03	853.8 ± 0.02
FA-CSF3	1562 ± 0.02	584.9 ± 0.01	791.4 ± 0.03
CSF	305.2 ± 0.00 *	753.5 ± 0.00 *	586.5 ± 0.00 *

* The significant difference between the groups at (*p < 0.05*).

## Data Availability

The outcome of data from this study.

## References

[1] Pan C., Chen S., Hao S., Yang X. (2019). Effect of low-temperature preservation on quality changes in Pacific white shrimp, Litopenaeus vannamei: A review. J. Sci. Food Agric..

[2] Qiu X., Tian H., Davis D.A. (2017). Evaluation of a high protein distiller’s dried grains product as a protein source in practical diets for Pacific white shrimp Litopenaeus vannamei. Aquaculture.

[3] Dayal J.S., Ponniah A.G., Khan H.I., Babu E.M., Ambasankar K., Vasagam K.K. (2013). Shrimps–a nutritional perspective. Curr. Sci..

[4] Gomez-Estaca J., Calvo M.M., Alvarez-Acero I., Montero P., Gomez-Guillen M.C. (2017). Characterization and storage stability of astaxanthin esters, fatty acid profile and α-tocopherol of lipid extract from shrimp (*L. vannamei*) waste with potential applications as food ingredient. Food Chem..

[5] Corral-Rosales C., Ricque-Marie D., Cruz-Suarez L.E., Arjona O., Palacios E. (2019). Fatty acids, sterols, phenolic compounds, and carotenoid changes in response to dietary inclusion of Ulva clathrata in shrimp Litopenaeus vannamei broodstock. J. Appl. Phycol..

[6] Olier B.S., Tubin J.S.B., de Mello G.L., Martínez-Porchas M., Emerenciano M.G.C. (2020). Does vertical substrate could influence the dietary protein level and zootechnical performance of the Pacific white shrimp Litopenaeus vannamei reared in a biofloc system?. Aquac. Int..

[7] Hamed I., Ozogul F., Regenstein J.M. (2016). Industrial applications of crustacean by-products (chitin, chitosan, and chitooligosaccharides): A review. Trends Food Sci. Technol..

[8] Trung T.S., Van Hoa N., Phuong P.T.D., Minh N.C., Mai N.T.H., Cuong H.N., Loc P.T., Stevens W.F. (2020). Recovery of Bioactive Components from Seafood By-Products toward Zero-Waste Processing.

[9] Shamshina J.L. (2019). Chitin in ionic liquids: Historical insights into the polymer’s dissolution and isolation. A review. Green Chem..

[10] Barber P.S., Griggs C.S., Bonner J.R., Rogers R.D. (2013). Electrospinning of chitin nanofibers directly from an ionic liquid extract of shrimp shells. Green Chem..

[11] Roy J.C., Salaün F., Giraud S., Ferri A., Chen G., Guan J. (2017). Solubility of chitin: Solvents, solution behaviors and their related mechanisms. Sol. Polysacch..

[12] Xia L., Wang S., Jiang Z., Chi J., Yu S., Li H., Zhang Y., Li L., Zhou C., Liu W. (2021). Hemostatic performance of chitosan-based hydrogel and its study on biodistribution and biodegradability in rats. Carbohydr. Polym..

[13] Pieklarz K., Galita G., Tylman M., Maniukiewicz W., Kucharska E., Majsterek I., Modrzejewska Z. (2021). Physico-chemical properties and biocompatibility of thermosensitive chitosan lactate and chitosan chloride hydrogels developed for tissue engineering application. J. Funct. Biomater..

[14] Narmani A., Jafari S.M. (2021). Chitosan-based nanodelivery systems for cancer therapy: Recent advances. Carbohydr. Polym..

[15] Jung H.Y., Le Thi P., HwangBo K.H., Bae J.W., Park K.D. (2021). Tunable and high tissue adhesive properties of injectable chitosan-based hydrogels through polymer architecture modulation. Carbohydr. Polym..

[16] Mukhtar M., Fenyes E., Bartos C., Zeeshan M., Ambrus R. (2021). Chitosan biopolymer, its derivatives and potential applications in nano-therapeutics: A comprehensive review. Eur. Polym. J..

[17] Mesgari M., Aalami A.H., Sahebkar A. (2021). Antimicrobial activities of chitosan/titanium dioxide composites as a biological nanolayer for food preservation: A review. Int. J. Biol. Macromol..

[18] Wang G., Li R., Parseh B., Du G. (2021). Prospects and challenges of anticancer agents’ delivery via chitosan-based drug carriers to combat breast cancer: A review. Carbohydr. Polym..

[19] Mostafa M., El-Meligy M.A., Sharaf M., Soliman A.T., AbuKhadra M.R. (2021). Insight into chitosan/zeolite-A nanocomposite as an advanced carrier for levofloxacin and its anti-inflammatory properties; loading, release, and anti-inflammatory studies. Biol. Macromol..

[20] Wei X., Ding S., Liu S., Yang K., Cai J., Li F., Wang C., Lin S., Tian F. (2021). Polysaccharides-modified chitosan as improved and rapid hemostasis foam sponges. Carbohydr. Polym..

[21] Torkaman S., Rahmani H., Ashori A., Najafi S.H.M. (2021). Modification of chitosan using amino acids for wound healing purposes: A review. Carbohydr. Polym..

[22] Florez M., Guerra-Rodriguez E., Cazon P., Vazquez M. (2022). Chitosan for food packaging: Recent advances in active and intelligent films. Food Hydrocolloid..

[23] Jhaveri J., Raichura Z., Khan T., Momin M., Omri A. (2021). Chitosan nanoparticles-insight into properties, functionalization and applications in drug delivery and theranostics. Molecules.

[24] Massarelli E., Silva D., Pimenta A.F.R., Fernandes A.I., Mata J.L.G., Armes H., Salema-Oom M., Saramago B., Serro A.P. (2021). Polyvinyl alcohol/chitosan wound dressings loaded with antiseptics. Int. J. Pharm..

[25] Vazquez M., Velazquez G., Cazon P. (2021). UV-Shielding films of bacterial cellulose with glycerol and chitosan. Part 2: Structure, water vapor permeability, spectral and thermal properties. CyTA-J. Food.

[26] Yee Kuen C., Masarudin M.J. (2022). Chitosan nanoparticle-based system: A new insight into the promising controlled release system for lung cancer treatment. Molecules.

[27] Okagu O.D., Jin J., Udenigwe C.C. (2021). Impact of succinylation on pea protein-curcumin interaction, polyelectrolyte complexation with chitosan, and gastrointestinal release of curcumin in loaded-biopolymer nano-complexes. J. Mol. Liq..

[28] Luque-Alcaraz A.G., Lizardi-Mendoza J., Goycoolea F.M., Higuera-Ciapara I., Argüelles-Monal W. (2016). Preparation of chitosan nanoparticles by nanoprecipitation and their ability as a drug nanocarrier. RSC Adv..

[29] Vaezifar S., Razavi S., Golozar M.A., Karbasi S., Morshed M., Kamali M. (2013). Effects of some parameters on particle size distribution of chitosan nanoparticles prepared by ionic gelation method. J. Clust. Sci..

[30] Koummich S.A., Zoukh I.M., Gorachinov F., Geskovski N., Makreski P., Dodov M.G., Goracinova K. (2021). Design of ophthalmic micelles loaded with diclofenac sodium: Effect of chitosan and temperature on the block-copolymer micellization behaviour. Drug Deliv. Transl. Res..

[31] Chen X., Fan M., Tan H., Ren B., Yuan G., Jia Y., Li J., Xiong D., Xing X., Niu X. (2019). Magnetic and self-healing chitosan-alginate hydrogel encapsulated gelatin microspheres via covalent cross-linking for drug delivery. Mater. Sci. Eng. C.

[32] Essa D., Choonara Y.E., Kondiah P.P., Pillay V. (2020). Comparative nanofabrication of PLGA-chitosan-PEG systems employing microfluidics and emulsification solvent evaporation techniques. Polymers.

[33] Silveira N.M., Prataviera P.J., Pieretti J.C., Seabra A.B., Almeida R.L., Machado E.C., Ribeiro R.V. (2021). Chitosan-encapsulated nitric oxide donors enhance physiological recovery of sugarcane plants after water deficit. Environ. Exp. Bot..

[34] Park S.J., Lee Y.K., Cho S., Uthaman S., Park I.K., Min J.J., Ko S.Y., Park J.O., Park S. (2015). Effect of chitosan coating on a bacteria-based alginate microrobot. Biotechnol. Bioeng..

[35] Ahmed F., Soliman F.M., Adly M.A., Soliman H.A., El-Matbouli M., Saleh M. (2019). Recent progress in biomedical applications of chitosan and its nanocomposites in aquaculture: A review. Res. Vet. Sci..

[36] Alterary S.S., Marei N.H. (2021). Fly ash properties, characterization, and applications: A review. J. King Saud Uni.-Sci..

[37] Szerement J., Szatanik-Kloc A., Jarosz R., Bajda T., Mierzwa-Hersztek M. (2021). Contemporary applications of natural and synthetic zeolites from fly ash in agriculture and environmental protection. J. Clean. Prod..

[38] Gadore V., Ahmaruzzaman M. (2021). Tailored fly ash materials: A recent progress of their properties and applications for remediation of organic and inorganic contaminants from water. J. Water Process Eng..

[39] Kanti P., Sharma K.V., CG R., Azmi W.H. (2021). Experimental determination of thermophysical properties of Indonesian fly-ash nanofluid for heat transfer applications. Part. Sci. Technol..

[40] Rodwihok C., Suwannakeaw M., Charoensri K., Wongratanaphisan D., Woo S.W., Kim H.S. (2021). Alkali/zinc-actvated fly ash nanocomposites for dye removal and antibacterial applications. Bioresour. Technol..

[41] Amariei G., Valenzuela L., Iglesias-Juez A., Rosal R., Visa M. (2022). ZnO-functionalized fly-ash based zeolite for ciprofloxacin antibiotic degradation and pathogen inactivation. J. Environ. Chem. Eng..

[42] Pakizeh M., Moradi A., Ghassemi T. (2021). Chemical extraction and modification of chitin and chitosan from shrimp shells. Eur. Polym. J..

[43] Marei N.H., El-Mazny W., El-Shaer A., Zaki K.D., Hussein Z.S., Abd-El-Samie E.M. (2017). Enhanced wound healing activity of desert locust (Schistocerca gregaria) vs. shrimp (Penaeus monodon) chitosan-based scaffolds. Int. J. Biol. Macromol..

[44] Gaballah S.T., El-Nazer H.A., Abdel-Monem R.A., El-Liethy M.A., Hemdan B.A., Rabie S.T. (2019). Synthesis of novel chitosan-PVC conjugates encompassing Ag nanoparticles as antibacterial polymers for biomedical applications. Int. J. Biol. Macromol..

[45] Alterary S., Marei N.H. (2020). The impact of coal fly ash purification on its antibacterial activity. Minerals.

[46] Souza V.G.L., Pires J.R.A., Rodrigues C., Rodrigues P.F., Lopes A., Silva R.J., Caldeira J., Duarte M.P., Fernandes F.B., Coelhoso I.M. (2019). Physical and morphological characterization of chitosan/montmorillonite films incorporated with ginger essential oil. Coatings.

[47] Kumari S., Kumar Annamareddy S.H., Abanti S., Kumar Rath P. (2017). Physicochemical properties and characterization of chitosan synthesized from fish scales, crab, and shrimp shells. Int. J. Biol. Macromol..

[48] Klancnik A., Piskernik S., Jersek B., Mozina S.S. (2010). Evaluation of diffusion and dilution methods to determine the antibacterial activity of plant extracts. J. Microbiol. Methods.

[49] Hisham F., Akmal M.M., Ahmad F.B., Ahmad K. (2021). Facile extraction of chitin and chitosan from shrimp shell. Mater. Today Proc..

[50] Czechowska-Biskup R., Jarosinska D., Rokita B., Ulanski P., Rosiak J.M. (2012). Determination of degree of deacetylation of chitosan-comparision of methods. Prog. Chem. Appl. Chitin Deriv..

[51] Fatoni A., Loekitowati P., Hermansyah H., Lesbani A. (2018). Synthesis and characterization of chitosan linked by methylene bridge and schiff base of 4, 4-diaminodiphenyl ether-vanillin. Indones. J. Chem..

[52] Queiroz M.F., Teodosio Melo K.R., Sabry D.A., Sassaki G.L., Rocha H.A.O. (2014). Does the use of chitosan contribute to oxalate kidney stone formation?. Mar. Drugs.

[53] Melo-Silveira R.F., Fidelis G.P., Costa M.S.S.P., Telles C.B.S., Dantas-Santos N., de Oliveira Elias S., Ribeiro V.B., Barth A.L., Macedo A.J., Leite E.L. (2011). In vitro antioxidant, anticoagulant and antimicrobial activity and in inhibition of cancer cell proliferation by xylan extracted from corn cobs. Int. J. Mol. Sci..

[54] Wolkers W.F., Oliver A.E., Tablin F., Crowe J.H. (2004). A Fourier-transform infrared spectroscopy study of sugar glasses. Carbohydr. Res..

[55] Silva F.R.F., Dore C.M.P.G., Marques C.T., Nascimento M.S., Benevides N.M.B., Rocha H.A.O., Chavante S.F., Leite E.L. (2010). Anticoagulant activity, paw edema and pleurisy induced carrageenan: Action of major types of commercial carrageenans. Carbohydr. Polym..

[56] Plessis P.W.D., Ojumu T.V., Petrik L.F. (2013). Waste minimization protocols for the process of synthesizing zeolites from South African coal fly ash. Materials.

[57] Morsy M., Mostafa K., Amyn H., El-Ebissy A.A.H., Salah A.M., Youssef M.A. (2019). Synthesis and Characterization of Freeze Dryer Chitosan Nano particles as Multi functional Eco-Friendly Finish for Fabricating Easy Care and Antibacterial Cotton Textiles. Egypt. J. Chem..

[58] Fenelonov V.B., Mel’gunov M.S., Parmon V.N. (2010). The properties of cenospheres and the mechanism of their formation during high-temperature coal combustion at thermal power plans. KONA Powder Part. J..

[59] Zhang Z.H., Han Z., Zeng X.A., Xiong X.Y., Liu Y.J. (2015). Enhancing mechanical properties of chitosan films via modification with vanillin. Int. J. Biol. Macromol..

[60] Abugoch L.E., Tapia C., Villaman M.C., Yazdani-Pedram M., Diaz-Dosque M. (2011). Characterization of quinoa protein–chitosan blend edible films. Food Hydrocoll..

[61] Hossain M.S., Iqbal A. (2014). Production and characterization of chitosan from shrimp waste. J. Bangl. Agr. Univ..

[62] Nessa F., Masum S.M., Asaduzzaman M., Roy S.K., Hossain M.M., Jahan M.S. (2010). A process for the preparation of chitin and chitosan from prawn shell waste. Bangl. J. Sci. Ind. Res..

[63] Gafri H.F.S., Zuki F.M., Aroua M.K., Hashim N.A. (2019). Mechanism of bacterial adhesion on ultrafiltration membrane modified by natural antimicrobial polymers (chitosan) and combination with activated carbon (PAC). Rev. Chem. Eng..

[64] Li J., Fu J., Tian X., Hua T., Poon T., Koo M., Chan W. (2022). Characteristics of chitosan fiber and their effects towards improvement of antibacterial activity. Carbohydr. Polym..

